# Supplementary material for: Circulating inflammatory proteins predict dementia risk, and are linked to structural brain changes and modifiable risk factors
Source: Alzheimers Res Ther. 2026 Jan 19;18:27. doi: 10.1186/s13195-025-01951-z (PMC12879394; doi:10.1186/s13195-025-01951-z)
Supplement: Supplementary file 1 — Supplementary Material 1 [file 13195_2025_1951_MOESM1_ESM.docx]

Supplementary methods

###

To explore whether ProSig or any individual ProSig protein mediated the effect of genetic (*APOE-ε4* allele count and an AD PRS) and modifiable risk factors (education, BMI, diabetes, hypertension, Townsend index, smoking and drinking status) on dementia risk, we conducted mediation analyses following the framework described by

Baron and Kenny and additionally estimated indirect effects.^1,2^ The Baron and Kenney criteria investigate the presence of a mediation effect using the following steps: (1) establish an association between the predictor X (*APOE-ε4*, AD PRS or lifestyle risk factors) and the outcome Y (incidence of dementia), (2) establish an association between the predictor X and the mediator M (ProSig or ProSig protein levels), (3) establish an association between the mediator M and the outcome Y while controlling for the predictor, and (4) observe a reduction or elimination of the association of the predictor and the outcome Y when the mediator M is included in the model, indicating partial or total mediation. In the first step, we used Cox-PH regression to assess the association between each risk factor (genetic or modifiable) and time to incident dementia. We then performed linear regressions between each risk factor and ProSig or individual protein levels. In step three we performed a Cox-PH regression investigating the association between genetic and modifiable risk factors, and proteins with time in years to the incidence of dementia. Protein coefficients were recorded while adjusting for each risk factor. Proteins that were not significantly associated with incident dementia after adjustment for the risk factors (*p* ≥ 0.05) were excluded from further analyses (step 3). As a fourth step, the coefficients for the association of the risk factors with time to incident dementia when adjusting for proteins were recorded. If the association with dementia was still significant after protein adjustment, and of the same magnitude as the association between dementia and the risk factor variables without protein adjustment, the mediation criteria were not met, and we concluded that the association of the risk factors with dementia was not mediated by these proteins. Mediation analyses were performed for each protein separately and all regression models were adjusted for age, age^2^, sex and their interaction terms (age$\times$sex, age^2^$\times$sex). If partial or full mediation was observed, the indirect effect of the risk factor was estimated using the product of coefficients method (path a $\times$ path b); the confidence intervals were obtained using bootstrapping with the R package “boot” (Version 1.3-28).^3^ The total effect was estimated as indirect effect + direct effect (step 3, coefficient c’ for the variables).

1 Baron RM, Kenny DA. The moderator–mediator variable distinction in social psychological research: Conceptual, strategic, and statistical considerations. *J Pers Soc Psychol* 1986; **51**: 1173–82.

2 VanderWeele TJ. Mediation Analysis: A Practitioner’s Guide. *Annu Rev Public Health* 2016; **37**: 17–32.

3 Davison AC, Hinkley D V. Bootstrap Methods and their Application. Cambridge University Press, 1997 DOI:10.1017/CBO9780511802843.
