# Supplementary material for: Circulating inflammatory proteins predict dementia risk, and are linked to structural brain changes and modifiable risk factors
Source: Alzheimers Res Ther. 2026 Jan 19;18:27. doi: 10.1186/s13195-025-01951-z (PMC12879394; doi:10.1186/s13195-025-01951-z)
Supplement: Supplementary file 3 — Supplementary Material 3 [file 13195_2025_1951_MOESM3_ESM.docx]

a


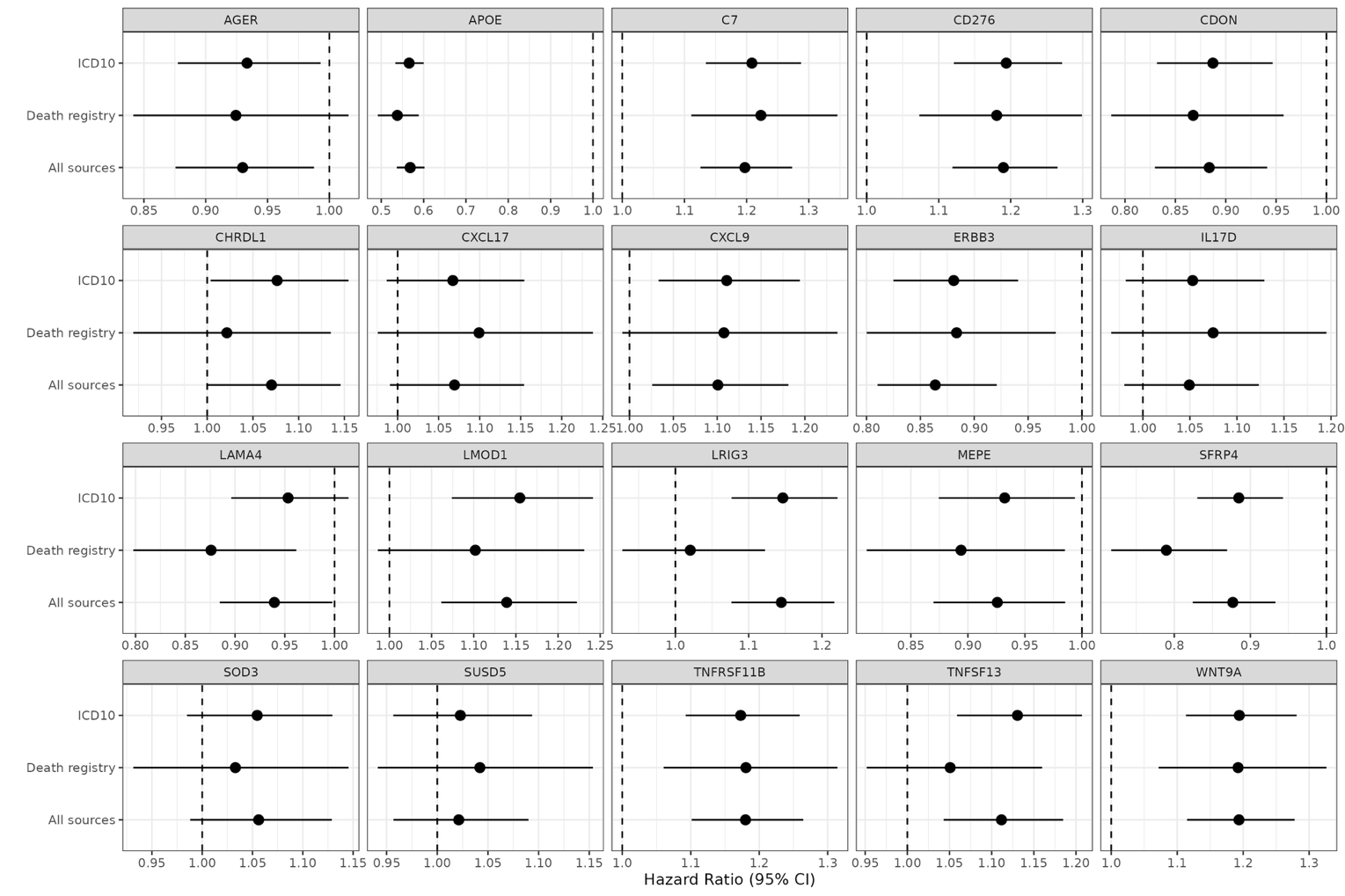


b


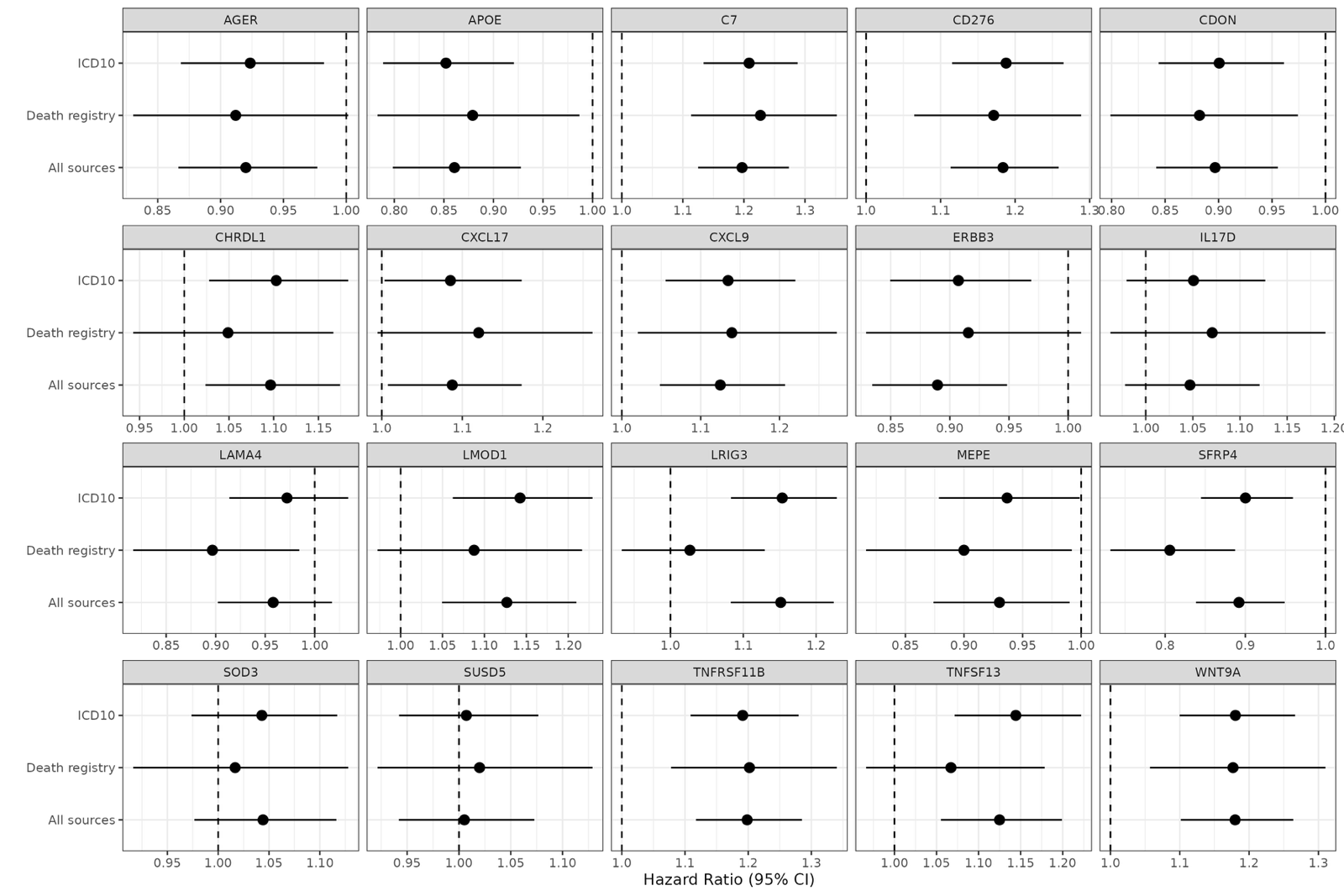


c


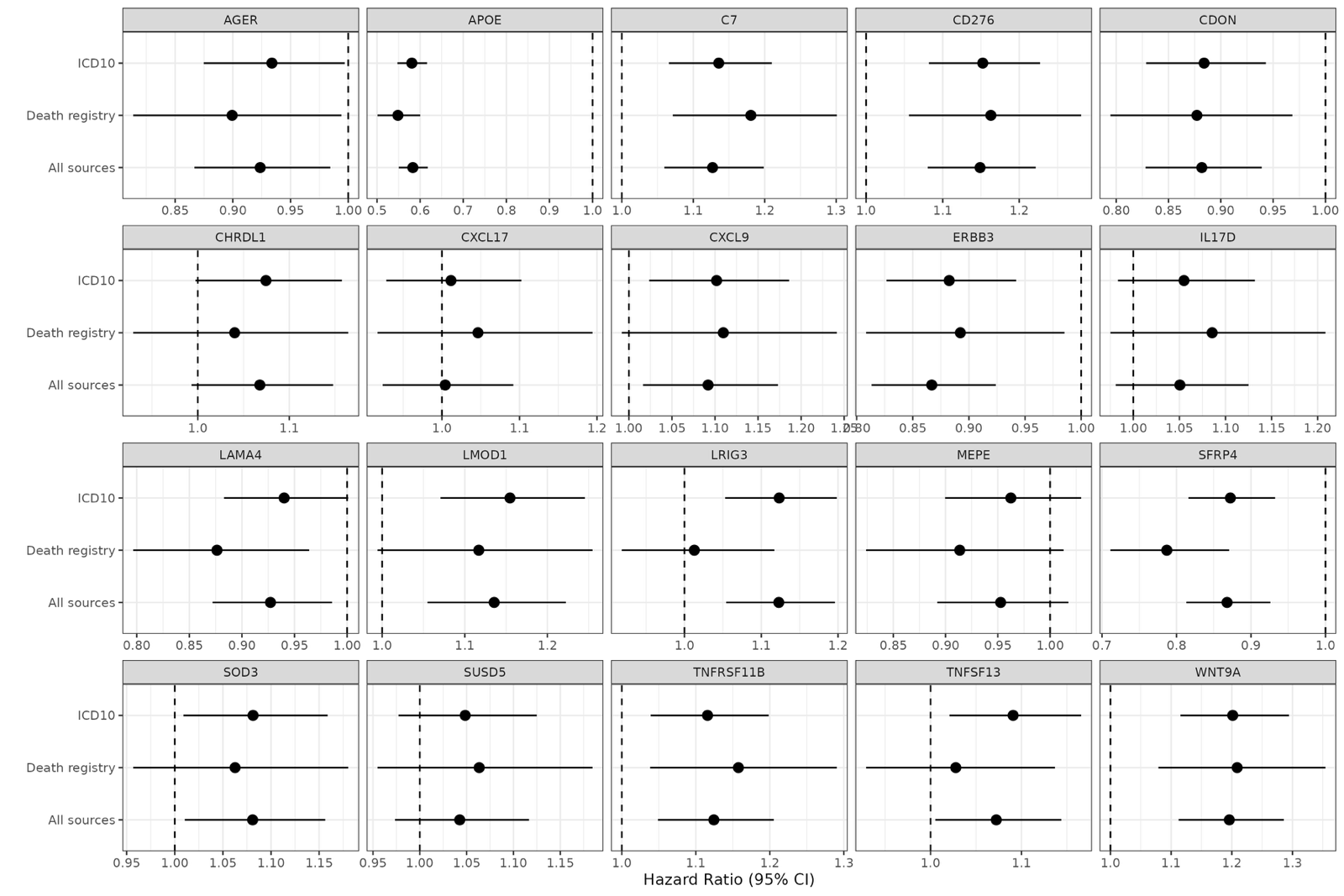


d


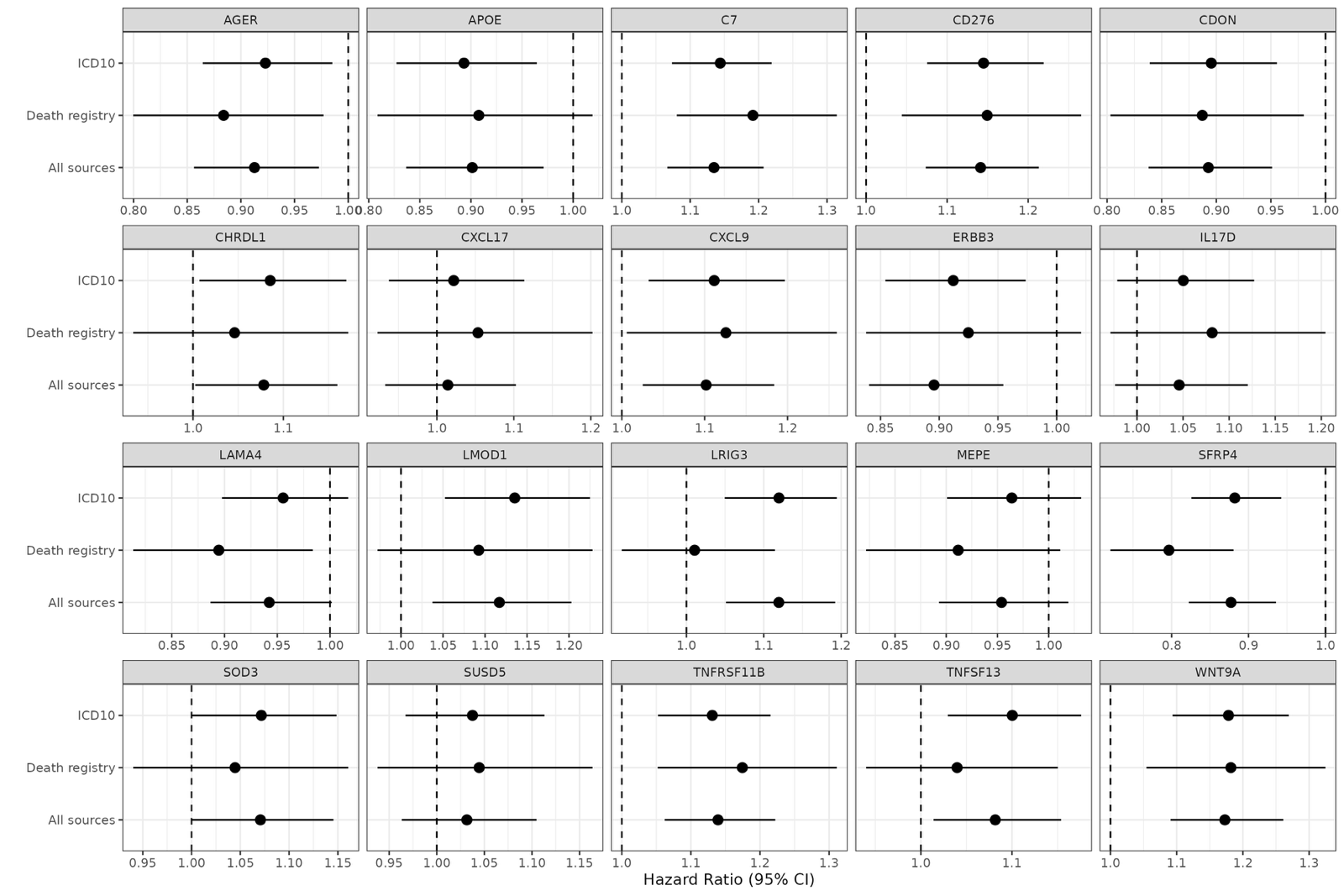


**Supplementary Figure 1.** **Cox proportional hazards models for ProSig proteins and dementia risk stratified by diagnostic source.** Association between individual ProSig proteins and incident dementia across four sequentially adjustment models (M1-M4). Models were adjusted for sex and age (M1; a), sex, age and APOE-ε4 alleles (M2; b), sex, age, ethnicity, eGFR and lifestyle risk factors (M3; c) and sex, age, ethnicity, eGFR and lifestyle risk factors and APOE-ε4 alleles (M4; d). The modifiable risk factors include BMI, education, deprivation, hypertension, diabetes, alcohol consumption and smoking status. Forest plots show hazard rations and the error bars indicate 95% confidence intervals. The dashed vertical line at HR = 1.0 indicates no association.

FDR < 0.05 *

FDR < 0.01 **

FDR < 0.001 ***

**Supplementary Figure 2. The association between proteomic signature (ProSig) proteins with global MRI-derived brain volumes and dementia subtypes.** The top panels show the associations between image-derived phenotypes (y-axis; outcome) and ProSig proteins (x-axis; predictor) using linear regressions. Each regression model for each image-derived phenotypes was adjusted for the number of proteins (n = 20). Bottom panels show the Cox PH-derived association of incident all-cause dementia (ACD), Alzheimer’s disease (AD) and vascular dementia (VaD) with ProSig proteins in all individuals with proteomic measurements who passed quality control (n = 43,685). Each regression model for each dementia outcome was adjusted for the total number of proteins in the inflammatory panel (n = 728). WB: whole brain; RH: right hemisphere; LH: left hemisphere.
